# Drift Eliminating Designs for Non-Simultaneous Comparison Calibrations

**DOI:** 10.6028/jres.098.016

**Published:** 1993

**Authors:** Ted Doiron

**Affiliations:** National Institute of Standards and Technology, Gaithersburg, MD 20899-0001

**Keywords:** calibration design, comparison, drift eliminating, least squares analysis, metrology

## Abstract

The effects of drift on calibrations carried out by comparison have been studied at the National Institute of Standards and Technology for many years, and a number of strategies have been introduced to combat these effects. One strategy, the use of comparison designs which are inherently immune to linear drift, was developed specifically for mass comparison measurements. These techniques, developed for simultaneous comparisons, are extended to the case of non-simultaneous comparisons, such as gage block calibrations, where each artifact is measured separately, and the comparison is made mathematically from the individual measurements.

## 1. Introduction

The sources of variation in measurements are numerous. Some of the sources are truly random noise, 1/*f* noise in electronic circuits for example. Usually the “noise” of a measurement is actually due to uncontrolled systematic effects such as instability of the mechanical setup or variations in the conditions or procedures of the test. Many of these variations are random in the sense that they are describable by a normal distribution. Like true noise in the measurement system, the effects can be reduced by making additional measurements.

Another source of serious problems, which is not random in nature is drift in the instrument readings. This effect cannot be minimized by additional measurement because it is not generally pseudorandom, but a nearly monotonie shift in the readings. In dimensional work the most important cause of drift is thermal changes in the equipment during the test. In this paper we will demonstrate techniques to address this problem of instrumental drift.

A simple example of the techniques for eliminating the effects of drift by looking at two different ways of comparing two gage blocks, one standard (A) and one unknown (B).

Scheme 1: A B A B
Scheme 2: A B B A

Now let us suppose we make the measurements regularly spaced in time, 1 time unit apart, and there is an instrumental drift of △. The actual readings (*m_i_*) from scheme 1 are:

$$m_{1}=A$$ (1a)

$$m_{2}=B+\Delta$$ (1b)

$$m_{3}=A+2\Delta$$ (1c)

$$m_{4}=B+3\Delta$$ (1d)

Solving for *B* in terms of *A* we get:

$$B=A-\frac{1}{2}\left(m_{1}+m_{3}-m_{2}-m_{4}\right)-\Delta$$ (2)

which depends on the drift rate *∆*.

Now look at scheme 2. Under the identical conditions the readings are:

$$m_{1}=A$$ (3a)

$$m_{2}=B+\Delta$$ (3b)

$$m_{3}=B+2\Delta$$ (3c)

$$m_{4}=A+3\Delta$$ (3d)

Here we see that if we add the second and third readings and subtract the first and fourth readings we find that the ∆ drops out:

$$B=A-\frac{1}{2}\left(m_{1}+m_{4}-m_{2}-m_{3}\right)$$ (4)

Thus if the drift rate is constant—a fair approximation for most measurements if the time is properly restricted—the analysis both eliminates the drift and supplies a numerical approximation of the drift rate.

The calibration of a small number of “unknown” objects relative to one or two reference standards involves determining differences among the group of objects. Instrumental drift, due most often to temperature effects, can bias both the values assigned to the objects and the estimate of the effect of random errors. This paper presents schedules for sequential measurements of differences that eliminate the bias from drift and at the same time gives estimates of the magnitude of drift.

Previous works have [1,2] discussed schemes which eliminate the effects of drift for simultaneous comparisons of objects. For these types of measurements the difference between two objects is determined at one instant of time. Examples of these types of measurements are comparisons of masses with a double pan balance, comparison of standard voltage cells, and thermometers which are all placed in the same thermalizing environment. Many comparisons, especially those in dimensional metrology, cannot be done simultaneously. For example, using a gage block comparator, the standard, control (check standard) and test blocks are moved one at a time under the measurement stylus. For these comparisons each measurement is made at a different time. Schemes which assume simultaneous measurements will, in fact, eliminate the drift from the analysis of the test objects but will produce a measurement variance which is drift dependent and an erroneous value for the drift, ∆.

Calibration designs involve differences between measured items so that unless one or more of them are standards for which values are known, one cannot assign values for the remaining “unknown” items. Algebraically, one has a system of equations that is not of full rank and needs the value for one item or the sum of several items as the restraint to lead to a unique solution. The least squares method used in solving these equations has been presented [3] and refined [4] in the literature and will not be repeated in detail here. The analyses of particular measurement designs presented later in this paper conform to the method and notation presented in detail by Hughes [3].

The schemes used as examples in this paper are those currently used at NIST for gage block comparisons. In our calibrations a control (check standard) is always used to generate data for our measurement assurance plan [5]. It is not necessary, however, to use a control in every design and the schemes can be used with any of the objects as the standard and the rest as unknowns. Schemes of various numbers of unknowns and measurements are presented in the Appendix.

## 2. Calibration Designs

The term calibration design has been applied to experiments where only differences between nominally equal objects or groups of objects can be measured. Perhaps the simplest such experiment consists in measuring the differences between the two objects of the *n* (*n* − 1) distinct pairings that can be formed from *n* objects. If the order is unimportant, *X* compared to *Y* is the negative of *Y* compared to *X*, and there are only *n* (*n* −1)/2 distinct pairings. Of course only one measurement per unknown is needed to determine the unknown, but many more measurements are generally taken for statistical reasons. Ordinarily the order in which these measurements are made is of no consequence. However, when the response of the comparator is time dependent, attention to the order is important if one wishes to minimize the effect of time.

When this effect can be adequately represented by a linear drift, it is possible to balance out the effect by proper ordering of the observations. The drift can be represented by the series,… −3, −2, −1, 0, 1, 2, 3, … if there are an odd number of comparisons and by … −5/2, −3/2, −1/2, 1/2, 3/2, 5/2,… if there are an even number of comparisons.

As an example let us take *n* =3. If we make 12 measurements to obtain all possible *n*(*n*−1) = 6 comparisons we get a scheme like that below, denoting the three objects by A, B, C.

$$\begin{array}{cc} \text{Observation} & \text{Measurement}\\ m_{1} & A-6\Delta \\ m_{2} & B-5\Delta \\ m_{3} & C-4\Delta \\ m_{4} & A-3\Delta \\ m_{5} & B-2\Delta \\ m_{6} & C-\;\Delta \\ m_{7} & A+\;\Delta \\ m_{8} & C+2\Delta \\ m_{9} & B+3\Delta \\ m_{10} & A+4\Delta \\ m_{11} & C+5\Delta \\ m_{12} & B+6\Delta \end{array}$$ (5)

If we analyze these measurements by pairs, in analogy to the weighing designs of Cameron we see that:

$$\begin{array}{ccccc} & A & B & C & \Delta \\ d_{1}=m_{1}-m_{2}=A-B-\Delta & +1 & -1 & & -1\\ d_{2}=m_{3}-m_{4}=C-A-\Delta & -1 & & +1 & -1\\ d_{3}=m_{5}-m_{6}=B-C-\Delta & & +1 & -1 & -1\\ d_{4}=m_{7}-m_{8}=A-C-\Delta & +1 & & -1 & -1\\ d_{5}=m_{9}-m_{10}=B-A-\Delta & -1 & +1 & & -1\\ d_{6}=m_{11}-m_{12}=C-B-\Delta & & -1 & +1 & -1 \end{array}$$ (6)

The notation used here, the plus and minus signs, indicate the items entering into the difference measurement. Thus, *d*_2_ is a measurement of the difference between object C and object A.

The difference between the above design and that of a design for simultaneous comparisons in Ref. [2] is that the drift column is constant. It is simple to see by inspection that the drift is balanced out since each object has two (+) and two (−) measurements and the drift column is constant. By extension, the effects of linear drift is eliminated in all complete block measurement schemes (those for which all objects are measured in all possible *n* (*n* − 1) combinations).

Although all schemes in which each object has equal numbers of (+) and (−) measurements is drift eliminating, there are practical criteria which must be met for the scheme to work. First, the actual drift must be linear. For dimensional measurements the instrument drift is usually due to changes in temperature. The usefulness of drift eliminating designs depends on the stability of the thermal environment and the accuracy required in the calibration. In the NIST gage block laboratory the environment is stable enough that the drift is linear at the 5 nm level over periods of 5 to 10 min. Our comparison plans are chosen so that the measurements can be made in this period.

Secondly, each measurement must be made in about the same amount of time so that the measurements are made at fairly regular intervals. In a completely automated system this is simple, but with human operators there is a natural tendency to make measurements simpler and quicker if the opportunity presents itself. For example, if the scheme has a single block measured two or more times in succession it is tempting to measure the object without removing it from the apparatus, placing it in its normal resting position, and returning it to the apparatus for the next measurement.

Finally, the measurements of each block are spread as evenly as possible across the design. Suppose in the scheme above where each block is measured four times block A is measured as the first measurement of *d*_1_, *d*_2_, *d*_3_, and *d*_4_. There is a tendency to leave block A near the measuring point rather than its normal resting position because it is used so often in the first part of the scheme. This allows block A to have a different thermal handling than the other blocks which can result in a thermal drift which is not the same as the other blocks.

## 3. Restraints

Because all of the measurements made in a calibration are relative comparisons, at least one value must be known to solve the system of equations. In the design of the last section, for example, if one has a single standard and two unknowns, the standard can be assigned to any one of the letters. (The same would be true of two standards and one unknown.) If there are two standards and one unknown, the choice of which pair of letters to assign for the standards can be important if all of the possible comparisons are not made. For full block designs (all possible comparisons are made) the uncertainty of the result does not depend on which letter is assigned to the standards or unknowns. For incomplete block designs the uncertainty of the results can depend on which letter the standard and unknowns are assigned. In these cases the customer blocks are assigned to minimize their variance and allow the larger variance for the measurement of the extra master (control).

This asymmetry occurs because every possible comparison between the four items has not been measured. For 4 objects there are 12 possible intercomparisons. If an eight measurement scheme is used all three unknowns cannot be compared directly to the standard the same number of times. For example, two unknowns can be compared directly with the standard twice, but the other unknown will have no direct comparisons. This indirect comparison to the standard results in a slightly larger variance for the block compared indirectly. Complete block plans, which compare each block to every other block equal number of times, has no such asymmetry, and thus removes any restriction on the measurement position of the control.

## 4. Example: 4 block, 12 comparison, Single Restraint Design for NIST Gage Block Calibration [4]

The gage block comparison scheme put into operation in 1989 consists of two standards blocks, denoted S and C, and two customer blocks to be calibrated, denoted Y and Z. In order to decrease the random error of the comparison process a new scheme was devised, consisting of all 12 possible comparisons between the four blocks. Because of continuing problems making penetration corrections, the scheme was designed to use either the S or C block as the restraint and the difference (*S* − *C*) as the control parameter. The S blocks are all steel, and are used as the restraint for all steel customer blocks. The C blocks are chrome carbide, and are used as the restraint for chrome and tungsten carbide blocks. The difference (*S* − *C*) is independent of the choice of restraint.

We chose a complete block scheme which assures that the design is drift eliminating and the blocks can be assigned to the letters of the design arbitrarily. We chose (*S* − *C*) as the first comparison. Since there are a large number of ways to arrange the 12 measurements for a complete block design, we added two restrictions as a guide to choose a “best” design.

1. It was decided to avoid schemes which measured the same block two or more times consecutively. There are many possible schemes where one or more blocks are measured twice consecutively. There is a great temptation to not remove and replace the blocks under these conditions. The analysis assumes that each measurement is made with the same motion and that the measurements are evenly spaced in time. Consecutive repetition threatens both these assumptions.
2. We decided that schemes in which the six measurements of each block were spread out as evenly as possible in time would be less likely to be affected by small non-linearities in the drift. For example, for some schemes measurements of one block are completed by the 8th comparison, leaving the final 1/3 of the comparisons with no sampling of that block.

The new scheme is as follows:

$$\begin{array}{rrrrrr} & S & C & Y & Z & \Delta \\ d_{1}=S-C & 1 & -1 & 0 & 0 & -1\\ d_{2}=Z-S & -1 & 0 & 0 & 1 & -1\\ d_{3}=Y-Z & 0 & 0 & 1 & -1 & -1\\ d_{4}=C-S & -1 & 1 & 0 & 0 & -1\\ d_{5}=C-Y & 0 & 1 & -1 & 0 & -1\\ D=d_{6}=Z-C & X=0 & -1 & 0 & 1 & 1\\ d_{7}=S-Y & 1 & 0 & -1 & 0 & -1\\ d_{8}=C-Z & 0 & 1 & 0 & -1 & -1\\ d_{9}=S-Z & 1 & 0 & 0 & -1 & -1\\ d_{10}=Y-C & 0 & -1 & 1 & 0 & -1\\ d_{11}=Y-S & -1 & 0 & 1 & 0 & -1\\ d_{12}=Z-Y & 0 & 0 & -1 & 1 & -1 \end{array}$$ (7)

When the *S* block is the restraint, with value *L*, the matrix equation to solve is:

$$\begin{array}{ccc} \left|\begin{array}{cc} X^{t}X & a\\ a^{t} & 0 \end{array}\right| & \left|\begin{array}{c} A\\ \lambda \end{array}\right| & =\left|\begin{array}{c} X^{t}Y\\ L \end{array}\right| \end{array}$$ (8)

where [*A^t^*] = [*S C Y Z* ⊿] is the vector to be estimated and [*a*^1^] = [1 0 0 0 0] is the restraint vector.

$$\left|\begin{array}{cc} X^{t}X & a\\ a^{t} & 0 \end{array}\right|=\left|\begin{array}{rrrrrr} 6 & -2 & -2 & -2 & 0 & 1\\ -2 & 6 & -2 & -2 & 0 & 0\\ -2 & -2 & 6 & -2 & 0 & 0\\ -2 & -2 & -2 & 6 & 0 & 0\\ 0 & 0 & 0 & 0 & 12 & 0\\ 1 & 0 & 0 & 0 & 0 & 0 \end{array}\right|$$ (9)

$$\left|\begin{array}{cc} B & h\\ a^{t} & 0 \end{array}\right|={\left|\begin{array}{cc} X^{t}X & a\\ a^{t} & 0 \end{array}\right|}^{-1}=\left(1/24\right)\left|\begin{array}{rrrrrr} 0 & 0 & 0 & 0 & 0 & 24\\ 0 & 6 & 3 & 3 & 0 & 24\\ 0 & 3 & 6 & 3 & 0 & 24\\ 0 & 3 & 3 & 6 & 0 & 24\\ 0 & 0 & 0 & 0 & 2 & 0\\ 24 & 24 & 24 & 24 & 0 & 0 \end{array}\right|$$ (10)

The estimate for *A* can be written as

$$<A>{=}^{\left|\begin{array}{cc} BX^{t} & h \end{array}\right|}\left|\begin{array}{c} D\\ L \end{array}\right|=1/24\left|\begin{array}{rrrrrrrrrrrrr} 0 & 0 & 0 & 0 & 0 & 0 & 0 & 0 & 0 & 0 & 0 & 0 & 24\\ -6 & 3 & 0 & 6 & 3 & -3 & -3 & 3 & -3 & -3 & 3 & -3 & 24\\ -3 & 3 & 3 & 3 & 3 & 0 & -6 & 0 & -3 & 3 & 6 & -3 & 24\\ -3 & 6 & -3 & 3 & 0 & 3 & -3 & -3 & -6 & 0 & 3 & 3 & 24\\ -2 & -2 & -2 & -2 & -2 & -2 & -2 & -2 & -2 & -2 & -2 & 2 & 0 \end{array}\right|$$ (11)

This leads to estimated values for *S*, *C*, *Y*, *Z*, and *Δ* which can be written as a function of the comparison measurements:

<S>=L (12a)

$$<C>=\left(1/8\right)\left(-2d_{1}+d_{2}+2d_{4}+d_{5}-d_{6}-d_{7}+d_{8}-d_{9}-d_{10}+d_{11}\right)+L$$ (12b)

$$<Y>=\left(1/8\right)\left(-d_{1}+d_{2}+d_{3}+d_{4}-d_{5}-2d_{7}-d_{9}+d_{10}+2d_{11}-d_{12}\right)+L$$ (12c)

$$<Z>=\left(1/8\right)\left(-d_{1}+2d_{2}-d_{3}+d_{4}+d_{6}-d_{7}-d_{8}-2d_{9}+d_{11}+d_{12}\right)+L$$ (12d)

$$<\Delta >=\left(-1/12\right)\left(d_{1}+d_{2}+d_{3}+d_{4}+d_{5}+d_{6}+d_{7}+d_{8}+d_{9}+d_{10}+d_{11}+d_{12}\right)+L$$ (12e)

The deviations of the measured values from the estimated values, ϵ_1_, *ϵ*_2_, …, *ϵ*_12_ can be determined from the equations above, or can be calculated directly using matrix methods. For example,

$$\in_{1}={\left(d_{1}\right)}_{\text{observed}}-(<S>-<C>).$$ (13)

These deviations provide the information needed to obtain a value, *s_w_*, which is the experimental estimate value for the short term process standard deviation, or within standard deviation *σ_w_*.

$$s^{2}=\sum_{i=1}^{n}\frac{{\delta_{i}}^{2}}{\left(n-m+1\right)}$$ (14)

The number of degrees of freedom, (*n*−*m* + 1), results from taking the number of observations (*n* = 12) less the number of unknowns (*m* =5; *S*, *C*, *Y*, *Z*, *Δ*), and then adding one for the restraint. Because of the complete block structure (all 12 possible combinations measured) the standard deviations of the predicted values for the three unknowns are the same:

$$s_{C}=s_{Y}=s_{Z}=\left(1/2\right)\;s.$$ (15)

The standard deviation of the predicted drift is

$$S_{\delta }=\sqrt{\frac{1}{12\;s}}.$$ (16)

## 5. Process Control

### 5.1 *F*–Test

Continued monitoring of the measurement process is required to assure that predictions based on the accepted values for process parameters are still valid. For gage block calibration at NIST, the process is monitored for precision by comparison of the observed standard deviation, *s_w_*, to the average of previous values, *σ_w_*. For this purpose the value of *s_w_* is recorded for every calibration done, and this data set is periodically analyzed to provide an updated value of the accepted process *σ_w_* for each gage block size.

The comparison is made using the *F* distribution, which governs the comparison of variances. The ratio of the variances *s*^2^ (derived from the model fit to each calibration) and $\sigma_{w}^{2}$ derived from the history is compared to the critical value *F* (8, ∞, *α*), which is the upper *α* probability point of the F distribution for degrees of freedom 8 and ∞. For calibrations at NIST, *α* is chosen as 0.01 to give *F*(8, ∞, .01) = 2.5. The condition to be checked is:

$$F=\frac{{s^{2}}_{\text{obs}}}{\sigma_{w}^{2}}<2.5.$$ (17)

If this condition is violated the calibration fails, and is repeated. If the calibration fails more than once the test blocks are re-inspected and the instrument checked and recalibrated. All calibrations, pass or fail, are entered into the history file.

### 5.2 *t*–Test

At NIST a control measurement is made with each calibration by using two known master blocks in each calibration. One of the master blocks is steel and the other chrome carbide. When a customer block is steel the steel master is used as the restraint, and when a customer block is carbide, the carbide master is used as the restraint. The use of a control measurement for calibrations is necessary in order to provide assurance of the continuing accuracy of the measurements. The *F*-test, while providing some process control, only attempts to control the repeatibility of the process, not the accuracy. The use of a control is also the easiest method for finding the long term variability of the measurement process [5].

While the use of a control in each calibration is not absolutely necessary, the practice is highly recommended. There are systems which use intermittent tests, e.g., measures a control set once a week [4]. This is a good strategy for automated systems because the chances of block to block operator errors is reduced greatly. For manual measurements the process variability, and of course the occurence of operator error is much higher.

The check for systematic error is given by comparison of the observed value of the difference between the standard and control blocks. If S is the standard it becomes the restraint, and if A is used as the control (*S*−*A*) is the control parameter for the calibration. This observed control is recorded for every calibration, and is used to periodically update the accepted, or average value, of the control. The process control step involves the comparison of the observed value of the control to the accepted (historical) value. The comparison is made using the Student *t*-distribution.

The control test demands that the observed difference between the control and its accepted value be less than 3 times the accepted long term standard deviation, *σ_t_*, of the calibration process. This value of the t-distribution implies that a good calibration will not be rejected with a confidence level of 99.7%. The condition to be checked is:

$$t=\frac{\left|{\left(<S>-<C>-(S-C\right)}_{acc}\right|}{\sigma_{1}}<3.$$ (18)

The value of *σ_t_* is obtained directly from the sequence of values of(< *S* > − < *C* >) arising in regular calibrations. The recorded (<*S* > − < *C* >) as the restraint, and < *S* > − < *C* > is again used as the control.

If both the precision (*F*-est) and accuracy (*t*-test) criteria are satisfied, the process is regarded as being “in control” and values for the unknowns, *Y* and *Z*, and their associated uncertainties are regarded as valid. Failure of either criterion is an “out-of-control” signal and the measurements are repeated.

The value for drift, (*Δ*), serves as an indicator of possible trouble if it changes markedly from its usual range of values. However, because any linear drift is balanced out, a change in the value does not of itself invalidate the result.

## 6. Conclusion

The choice of the order of comparisons is an important facet of calibrations, in particular if chosen properly the comparison scheme can be made immune to linear drifts in the measurement equipment. The idea of making a measurement scheme robust is a powerful one. What is needed to implement the idea is an understanding of the sources of variability in the measurement system. While such a study is sometimes difficult and time consuming because of the lack of reference material concerning many fields of metrology, the NIST experience has been that such efforts are rewarded with measurement procedures which, for about the same amount of effort, produce higher accuracy.

## 7. Appendix A. Selection of Other Drift Eliminating Designs

The following designs can be used with or without a control block. The standard block is denoted *S*, and the unknown blocks *A*, *B*, *C*, etc. If a check standard block is used it can be assigned to any of the unknown block positions. The name of the design is simply the number of blocks in the design and the total number of comparisons.

**Table:**

3–6 Design (One master block, 2 unknowns 4 measurements each)		
*y*_1_	=	*S* − *A*
*y*_2_	=	*B* − *S*
*y*_3_	=	*A* − *B*
*y*_4_	=	*A* − *S*
*y*_5_	=	*B* − *A*
*y*_6_	=	*S* − *B*

**Table:**

3–9 Design (One master block, 2 unknowns 6 measurements each)		
*y*_1_	=	*S* − *A*
*y*_2_	=	*B* − *A*
*y*_3_	=	*S* − *B*
*y*_4_	=	*A* − *S*
*y*_5_	=	*B* − *S*
*y*_6_	=	*A* − *B*
*y*_7_	=	*A* − *S*
*y*_8_	=	*B* − *A*
*y*_9_	=	*S* − *B*

**Table:**

4–8 Design (One master block, 3 unknowns 4 measurements each)		
*y*_1_	=	*S* − *A*
*y*_2_	=	*B* − *C*
*y*_3_	=	*C* − *S*
*y*_4_	=	*A* − *B*
*y*_5_	=	*A* − *S*
*y*_6_	=	*C* − *B*
*y*_7_	=	*S* − *C*
*y*_8_	=	*B* − *A*

**Table:**

4–12 Design (One master block, 3 unknowns 6 measurements each)		
*y*_1_	=	*S* − *A*
*y*_2_	=	*C* − *S*
*y*_3_	=	*B* − *C*
*y*_4_	=	*A* − *S*
*y*_5_	=	*A* − *B*
*y*_6_	=	*C* − *A*
*y*_7_	=	*S* − *B*
*y*_8_	=	*A* − *C*
*y*_9_	=	*S* − *C*
*y*_10_	=	*B* − *A*
*y*_11_	=	*B* − *S*
*y*_12_	=	*C* − *B*

**Table:**

5–10 Design (One master block, 4 unknowns 4 measurements each)		
*y*_1_	=	*S* − *A*
*y*_2_	=	*D* − *C*
*y*_3_	=	*S* − *B*
*y*_4_	=	*D* − *A*
*y*_5_	=	*C* − *B*
*y*_6_	=	*A* − *C*
*y*_7_	=	*B* − *S*
*y*_8_	=	*B* − *D*
*y*_9_	=	*C* − *S*
*y*_10_	=	*A* − *D*

**Table:**

6 – 12 Design (One master block, 5 unknowns 4 measurements each)		
*y*_1_	=	*S* − *A*
*y*_2_	=	*D* − *C*
*y*_3_	=	*E* − *B*
*y*_4_	=	*E* − *D*
*y*_5_	=	*C* − *A*
*y*_6_	=	*B* − *C*
*y*_7_	=	*S* − *E*
*y*_8_	=	*A* − *D*
*y*_9_	=	*A* − *B*
*y*_10_	=	*D* − *S*
*y*_11_	=	*B* − *E*
*y*_12_	=	*C* − *S*

**Table:**

7–14 Design (One master block, 4 unknowns 6 measurements each)		
*y*_1_	=	*S* − *A*
*y*_2_	=	*E* − *C*
*y*_3_	=	*B* − *D*
*y*_4_	=	*A* − *F*
*y*_5_	=	*S* − *E*
*y*_6_	=	*D* − *B*
*y*_7_	=	*A* − *C*
*y*_8_	=	*B* − *F*
*y*_9_	=	*D* − *E*
*y*_10_	=	*F* − *S*
*y*_11_	=	*E* − *A*
*y*_12_	=	*C* − *B*
*y*_13_	=	*C* − *S*
*y*_14_	=	*F* − *D*

**Table:**

8–16 Design (One master block, 7 unknowns 4 measurements each)		
*y*_1_	=	*S* − *A*
*y*_2_	=	*E* − *G*
*y*_3_	=	*F* − *C*
*y*_4_	=	*D* − *S*
*y*_5_	=	*B* − *E*
*y*_6_	=	*G* − *F*
*y*_7_	=	*C* − *B*
*y*_8_	=	*E* − *A*
*y*_9_	=	*F* − *D*
*y*_10_	=	*C* − *S*
*y*_11_	=	*A* − *G*
*y*_12_	=	*D* − *B*
*y*_13_	=	*C* − *S*
*y*_14_	=	*G* − *C*
*y*_15_	=	*B* − *D*
*y*_16_	=	*A* − *F*

**Table:**

9 – 18 Design (One master block, 7 unknowns 4 measurements each)		
*y*_1_	=	*S* − *A*
*y*_2_	=	*H* − *F*
*y*_3_	=	*A* − *B*
*y*_4_	=	*D* − *C*
*y*_5_	=	*E* − *G*
*y*_6_	=	*C* − *A*
*y*_7_	=	*B* − *F*
*y*_8_	=	*G* − *H*
*y*_9_	=	*D* − *S*
*y*_10_	=	*C* − *E*
*y*_11_	=	*H* − *S*
*y*_12_	=	*G* − *D*
*y*_13_	=	*C* − *S*
*y*_14_	=	*A* − *C*
*y*_15_	=	*F* − *D*
*y*_16_	=	*S* − *H*
*y*_17_	=	*E* − *B*
*y*_18_	=	*F* − *G*

**Table:**

10 – 20 Design (One master block, 9 unknowns 4 measurements each)		
*y*_1_	=	*S* − *A*
*y*_2_	=	*F* − *G*
*y*_3_	=	*I* − *C*
*y*_4_	=	*D* − *E*
*y*_5_	=	*A* − *H*
*y*_6_	=	*B* − *C*
*y*_7_	=	*G* − *H*
*y*_8_	=	*I* − *S*
*y*_9_	=	*E* − *F*
*y*_10_	=	*H* − *I*
*y*_11_	=	*D* − *F*
*y*_12_	=	*A* − *B*
*y*_13_	=	*C* − *I*
*y*_14_	=	*H* − *E*
*y*_15_	=	*B* − *G*
*y*_16_	=	*S* − *D*
*y*_17_	=	*F* − *B*
*y*_18_	=	*C* − *D*
*y*_19_	=	*G* − *S*
*y*_20_	=	*E* − *A*

**Table:**

11–22 Design (One master block, 10 unknowns 4 measurements each)		
*y*_1_	=	*S* − *A*
*y*_2_	=	*D* − *E*
*y*_3_	=	*G* − *I*
*y*_4_	=	*C* − *H*
*y*_5_	=	*A* − *B*
*y*_6_	=	*I* − *J*
*y*_7_	=	*H* − *F*
*y*_8_	=	*D* − *S*
*y*_9_	=	*B* − *C*
*y*_10_	=	*S* − *E*
*y*_11_	=	*A* − *G*
*y*_12_	=	*F* − *B*
*y*_13_	=	*E* − *F*
*y*_14_	=	*J* − *A*
*y*_15_	=	*C* − *D*
*y*_16_	=	*H* − *J*
*y*_17_	=	*F* − *G*
*y*_18_	=	*I* − *S*
*y*_19_	=	*B* − *H*
*y*_20_	=	*G* − *D*
*y*_21_	=	*J* − *C*
*y*_22_	=	*E* − *I*

The choice of the order of comparisons is an important facet of calibrations, in particular if chosen properly the comparison scheme can be made immune to linear drifts in the measurement equipment. The idea of making a measurement scheme robust is a powerful one. What is needed to implement the idea is an understanding of the sources of variability in the measurement system. While such a study is sometimes difficult and time consuming because of the lack of reference material concerning many fields of metrology, the NIST experience has been that such efforts are rewarded with measurement procedures which, for about the same amount of effort, produce higher accuracy.
